# Concept of an upright wearable positron emission tomography imager in humans

**DOI:** 10.1002/brb3.530

**Published:** 2016-08-05

**Authors:** Christopher E. Bauer, Julie Brefczynski‐Lewis, Gary Marano, Mary‐Beth Mandich, Alexander Stolin, Peter Martone, James W. Lewis, Gangadhar Jaliparthi, Raymond R. Raylman, Stan Majewski

**Affiliations:** ^1^ Center for Advanced ImagingWest Virginia UniversityMorgantownWVUSA; ^2^ Department of RadiologyWest Virginia UniversityMorgantownWVUSA; ^3^ Center for NeuroscienceWest Virginia UniversityMorgantownWVUSA; ^4^ Department of Physiology and PharmacologyWest Virginia UniversityMorgantownWVUSA; ^5^ Division of Physical TherapyDepartment of Human PerformanceWest Virginia UniversityMorgantownWVUSA; ^6^ Department of Neurobiology and AnatomyWest Virginia UniversityMorgantownWVUSA; ^7^ Department of Radiology and Medical ImagingUniversity of VirginiaCharlottesvilleVAUSA

**Keywords:** brain imaging, functional imaging, mobile imaging, molecular imaging, positron emission tomography, upright imaging

## Abstract

**Background:**

Positron Emission Tomography (PET) is traditionally used to image patients in restrictive positions, with few devices allowing for upright, brain‐dedicated imaging. Our team has explored the concept of wearable PET imagers which could provide functional brain imaging of freely moving subjects. To test feasibility and determine future considerations for development, we built a rudimentary proof‐of‐concept prototype (Helmet_PET) and conducted tests in phantoms and four human volunteers.

**Methods:**

Twelve Silicon Photomultiplier‐based detectors were assembled in a ring with exterior weight support and an interior mechanism that could be adjustably fitted to the head. We conducted brain phantom tests as well as scanned four patients scheduled for diagnostic F^18‐^ FDG PET/CT imaging. For human subjects the imager was angled such that field of view included basal ganglia and visual cortex to test for typical resting‐state pattern. Imaging in two subjects was performed ~4 hr after PET/CT imaging to simulate lower injected F^18‐^ FDG dose by taking advantage of the natural radioactive decay of the tracer (F^18^ half‐life of 110 min), with an estimated imaging dosage of 25% of the standard.

**Results:**

We found that imaging with a simple lightweight ring of detectors was feasible using a fraction of the standard radioligand dose. Activity levels in the human participants were quantitatively similar to standard PET in a set of anatomical ROIs. Typical resting‐state brain pattern activation was demonstrated even in a 1 min scan of active head rotation.

**Conclusion:**

To our knowledge, this is the first demonstration of imaging a human subject with a novel wearable PET imager that moves with robust head movements. We discuss potential research and clinical applications that will drive the design of a fully functional device. Designs will need to consider trade‐offs between a low weight device with high mobility and a heavier device with greater sensitivity and larger field of view.

## 1 Introduction

There has been an intense private and public interest within the neuroscience research community in developing a “next generation” human brain imager that can address some of the constraints and limitations of current imaging modalities (website‐a). One important limitation to overcome is the ability to image during upright, natural movements such as those characterizing certain disorders as well as required in human behavioral tasks. It is not likely that high‐resolution fMRI will be able to tolerate the disturbance in the B field caused by anything beyond minor head movement, and the superconducting magnets currently necessary to create a stable Tesla‐range B field are extremely large and bulky. Limited progress has been made in upright imagers that allow a patient to be seated, including an upright head‐only PET scanner (website‐b), a whole‐body upright MRI scanner (website‐c), dense array EEG, and near‐infrared spectroscopy (NIRS) imaging. However, none of these modalities currently combine high spatial resolution with whole‐brain imaging of a moving patient; neither current upright PET, magnetoencephalography (MEG), or MRI allow for large‐scale head motion, and the low field strength (0.6 Tesla) of the Fonar upright MRI scanner is prohibitive for fMRI as well as requiring one to be still during the actual imaging. EEG can tolerate some degree of motion, but has lower spatial resolution and cannot image the deep limbic structures critical to many types of tasks.

The study of naturally occurring motor activity with neuroimaging has thus been largely limited to small movements such as finger tapping in which the head can be kept very still, although some rare exceptions such as an MRI‐compatible supine exercise bike have been created (Mehta, Verber, Wieser, Schmit, & Schindler‐Ivens, 2012), further underscoring the need for motion freedom in this niche area of research. One important example is in the study of vestibular processes, which requires the complex integration of vestibular inputs with somato‐proprioception and visual information (Mast, Preuss, Hartmann, & Grabherr, 2014). Such studies have been severely limited, despite large populations of patients having balance disorders. In addition, cognitive, affective, social, and sensory processes are active experiences in which one must be upright to move and interact naturally with the environment. The deep brain structures that mediate affective and social experience and behaviors, such as amygdala, basal ganglia, and hippocampus are largely off‐limits to existing motion‐tolerant imaging technology such as EEG and NIRS.

**Figure 1:**
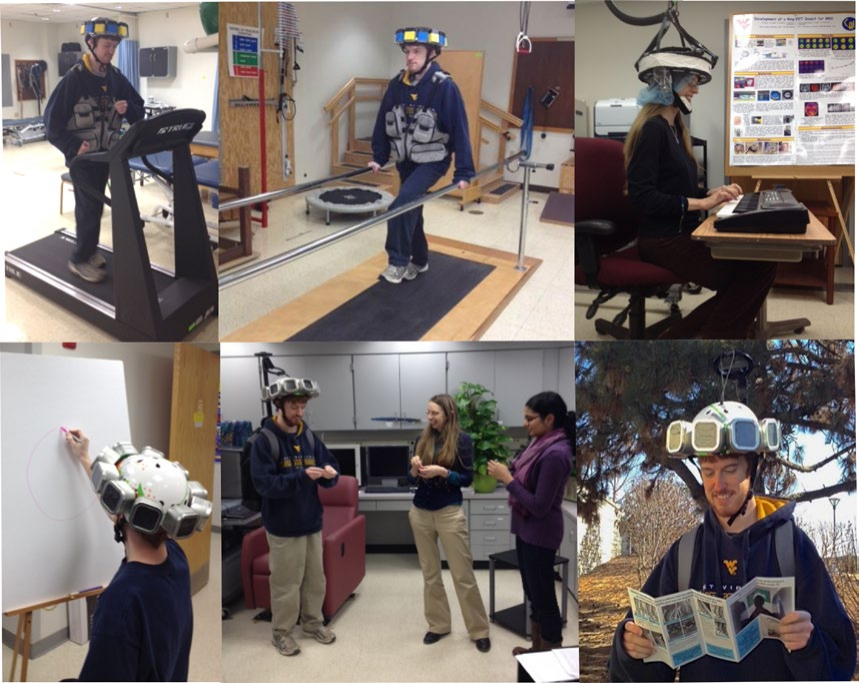
Concept of a future ambulatory Helmet_PET with freedom of movement. These six panels demonstrate activities that could be monitored by a mobile device. Specifically, motor tasks, artistic/creative tasks, and complex social interactions could be more easily studied

In this study we propose using compact PET detectors with silicon photomultiplier technology (SiPMs) to image the brain during both head and body motion by mounting these detectors in a ring or helmet which could move with the head (Fig. 1). Positron emission tomography (PET) is a well‐established molecular imaging modality and is considered to be one of the first brain imaging tools to allow high spatial resolution whole‐brain imaging, as it overcame the limitations of spatial resolution and surface‐only imaging of electroencephalography (EEG) (Mazziotta, 1985; Stephan et al., 1987). Behavioral studies showing whole‐brain activation patterns can be performed using markers of blood volume changes with O^15^‐H_2_O or metabolic changes with F^18^‐FDG. In addition, at higher spatial resolutions than MR spectroscopy, PET can image activity related to specific neurotransmitter systems, by using radiolabeled ligand targets such as D2 dopamine receptor antagonist C^11^‐raclopride (Carson et al., 1997; Kuhn et al., 2014), serotonin transporter binding agents C^11^‐Madam and C^11^‐Zient (Karlsson et al., 2013; Nye et al., 2013), and other ligands specific for opioids, as well as other targets (Weerts et al., 2012; Wey et al., 2014). Thus, a wearable PET scanner could be used in a variety of ways to uncover the mechanisms underlying behaviors, as well as psychological and neurological disorders.

A wearable PET device, if viable, would have significant application in the fields of neuroscience and mobile medicine (Fig. 1). In particular, there are two main advantages of such a device. First, this imager would overcome the high dose barrier by placing the detectors much closer to the brain. Low dose would enable more studies of the normal health brain, as well as longitudinal studies with repeated scans. Second, it would enable imaging of active, moving participants, thereby overcoming the stationary barrier. An ideal imager would be relatively lightweight, require a very low dose of ligand, and have mechanical support that can allow a considerable degree of free motion.

There are many functional processes to study in the human brain that can benefit from freedom of motion. Clinically, target populations which might benefit from such an imager include any group that has difficultly avoiding head motion (causing motion artifacts in a standard imager), such as patients with basal ganglia disease, Parkinson's disease, Alzheimer's disease, Epilepsy, Attention Deficit Disorder (ADD), and Autism Spectrum Disorder (ASD). A whole‐brain wearable imager would also enable imaging of individuals engaged in active natural behaviors and with those exceptional abilities such as artists and those with savant syndrome (Corrigan, Richards, Treffert, & Dager, 2012). Researchers and clinicians could also image brain changes with rehabilitation after a neurologic insult, such as traumatic brain injury (TBI) or stroke, allowing an individual's brain processes to guide selected treatment, rehabilitation strategy, or dose.

Only a few dedicated compact PET imagers have been developed. Several stationary versions have been created for human brain imaging (Olesen et al., 2009; Ouyang et al., 2012; Robertson, Marr, Rosenblum, Radeka, & Yamamoto, 1972; van Velden et al., 2009), with Photo Diagnostics' NeuroPET/CT being the first to use the compact Silicon Photomultiplier (SiPM) technology in a commercial mobile brain imager, however, this model is currently restricted to the supine position. A couple of devices allow some degree of upright head motion. The RatCap (Schulz & Vaska, 2011; Vaska et al., 2004) was created as a wearable rat brain PET imager using Avalanche Photodiode (APD) detectors that permitted real‐time collection of data in a rat moving in a restricted space. PET‐Hat, developed by Yamamoto et al., also allows some degree of head movement. The latter utilized a single ring of 16 detector modules based on position‐sensitive photomultiplier tubes (PSPMTs). Although compact in comparison to standard photomultiplier tubes (PMTs), PSPMTs are still bulky enough that the combined size and weight of the resulting PET‐Hat ring imager required a sophisticated counterbalanced mechanical support to allow the ring to follow only small movements of the patient's head, and thus far has only been tested with phantoms (Yamamoto, Honda, Oohashi, Shimizu, & Senda, 2011; Yamamoto, Honda, Shimizu, & Senda, 2009).

To test the feasibility of a truly wearable human PET imager, we obtained compact SiPM detectors and matching pixelated arrays of Cerium doped Lutetium Yttrium Orthosilicate (LYSO) scintillators and created a ring just large enough to fit around an adult head and acquire several axial slices of the brain. In particular, this device was built to include midline structures so we could test whether the typical patterns of high FDG signal intensity in structures like the caudate, thalamus, and visual cortex typically seen in resting PET were present in our images. We discuss the results of this proof‐of‐concept device on phantoms and humans, how these results will inform future design of a full device, and the potential implications and applications of a fully developed wearable imager in neuroscience research and in the clinic.

## 2 Method

### 2.1 proof‐of‐concept PET ring imager

The detailed description of the construction of this compact PET ring imager will be described in a separate technical communication, but in summary this prototype benefits from the very compact Hamamatsu Photonics (Hamamatsu City, Japan) Multi‐Pixel Photon Counter (MPPC) solid‐state Silicon Photomultiplier technology (website‐d) coupled to arrays of LYSO crystals with 1.5‐mm pitch and 10‐mm thickness from Proteus (Chagrin Falls, OH) (website‐e). Twelve modules of about 5 cm × 5 cm coverage (32 × 32 crystal pixels) are arranged in a ring geometry with ~21 cm inner diameter. Outside of a thin foam cushion and plastic frame to mount the head inside the imager, there is no material between the patient and the scintillators. A charge division readout from AiT Instruments (Newport News, VA, USA) (website‐f) that employs four readout channels per module was implemented. The 48 amplified detector signals were digitized with AiT Instruments' FPGA‐based 64 channel data acquisition (DAQ) module (Proffitt et al., 2005, 2006). A 16‐channel Mesytec (website‐g) MCFD‐16 coincidence module produced a trigger signal for the DAQ module when a coincidence was detected between any pair of modules in the ring. Readout software was implemented using the Java programming language (McKisson, Hammond, Proffitt, & Weisenberger, 2007) with an overlaying user interface provided by a Kmax scientific programming package from Sparrow Corporation (Port Orange, FL, USA) (website‐h).

As our goal was to assess initial feasibility, the device was kept simple, with no Time of Flight or Depth of Interaction advances applied. A 5‐cm‐wide ring allowed enough brain coverage to test for typical F^18‐^FDG patterns in midline brain structures.

**Figure 2:**
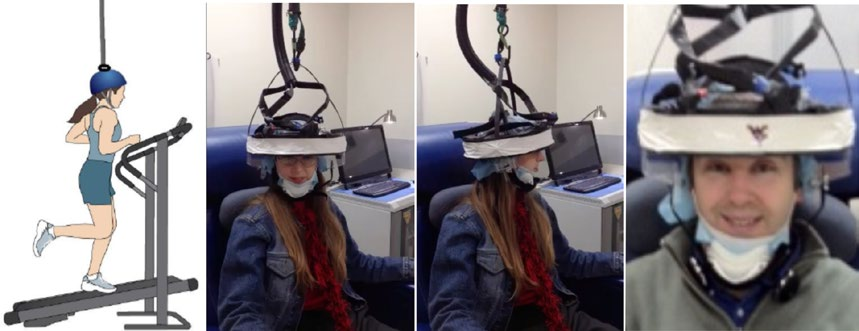
(Left) Toward the concept of a fully ambulatory imager within the laboratory environment; example: a running subject on a treadmill. Ultimately, this type of imager is what we hope is achieved. (Center Frames) Helmet_PET mounted on the head of a seated researcher and demonstrating freedom of rotational head movement by wide angles (J.B‐L.). This pilot imager was built to demonstrate the feasibility of a wearable PET imager (like the one illustrated on the left) using a single ring of detectors. (Right) Close up view of Helmet_PET showing the wearable and comfortable nature of this prototype (demonstrated by PM)

The ring of 12 detector modules suspended from above using a flexible mechanical mount with an overhead cable and rotating pulley system (Figs 2, S1). The ring could be adjusted vertically on the subject's head to image the desired section of the human brain (a fully developed model with more detectors would cover the whole brain). We were able to attach the imager to the patient's head using the adjustable plastic frame and a chin strap from a standard safety helmet. Helmet_PET is currently about 3 kg in weight, and this weight was supported by the overhead flexible cable and not perceived by the patient, allowing for limited movement.

### 2.2 System key parameters

The coincidence timing window used in acquisition was 10 ns, whereas the energy window used was approximately 400–600 keV for all measurements. The rate capability for this system was limited to about 47 kHz (activity level in the field of view = ~300 μCi). The spatial resolution at the center of the detector was approximately 2‐mm FWHM in the tangential direction, and about 2.8 mm in the radial direction. The absolute efficiency at the center of the imager was 0.7%, reflecting its lightweight small size.

### 2.3 Reconstruction and image processing

Images were reconstructed using a custom‐written MLEM (Maximum‐Likelihood Expectation‐Maximization) software algorithm (Unpublished). Reconstruction software for the ring PET imager was developed based on an algorithm previously developed for a two‐module PET system (Smith, Raylman, Majewski, & Weisenberger, 2004). Our reconstructions in the performed phantom evaluation experiments generally used 1 × 1 × 1 millimeter isotropic voxels and iterative reconstruction for 10 iterations, and an attenuation correction assuming the entire volume inside the imager is water was built into our reconstruction program. Images in the human imaging section involved reconstruction using 2 × 2 × 2 millimeter isotropic voxels, and resulted in 2‐mm‐thick brain slices. Reconstructed images were displayed using ImageJ public domain imaging software and the MIM professional software when comparing the Helmet_PET images with the PET/CT images (website‐i). Images for the human data were blurred with approximately 2‐mm Gaussian filtering and the smoothing algorithm native to ImageJ.

### 2.4 Uniformity calibration and “flood” correction

A simplified uniformity image correction method was used which is performed by image division of the reconstructed 2D slices obtained from the imaged object (such as a phantom or a patient brain) by the corresponding experimentally obtained slice images of a cylindrical “flood” phantom (a cylinder covering the entire useful field of view of the imager, which is filled with an F18‐FDG water solution with a uniform volume concentration of radioactivity). This uniformity correction accounts for not only the geometrical response matrix of the detectors but also for the bulk of the 511 keV annihilation gamma absorption effects.

A 185‐mm‐diameter thin‐wall acrylic cylinder was placed inside the PET ring and filled with an F^18‐^FDG water solution of uniform concentration while data were collected for 4 hr to attain high statistics images. The images of the brain phantoms and patients were collected for much shorter times. Although the reconstructed images that resulted from the brain phantom and patient data collection showed some nonuniformities, we verified that the division of the object images by the flood images obtained with the aforementioned 185‐mm flood phantom resulted in images which were much more uniform with a greatly reduced number of artifacts compared to uncorrected images.

### 2.5 Phantom imaging

We studied the operation of our prototype imager with many different phantoms of various sizes, but here we will only reproduce a few key examples of images obtained with a multicompartmental brain phantom (Data Spectrum Corp, Product Code BR/2D‐MC/P) (website‐j). The images were reconstructed with reconstruction‐encoded attenuation correction and then “normalized” (as described above) by the system response from a uniform cylinder “flood” phantom.

**Figure 3:**
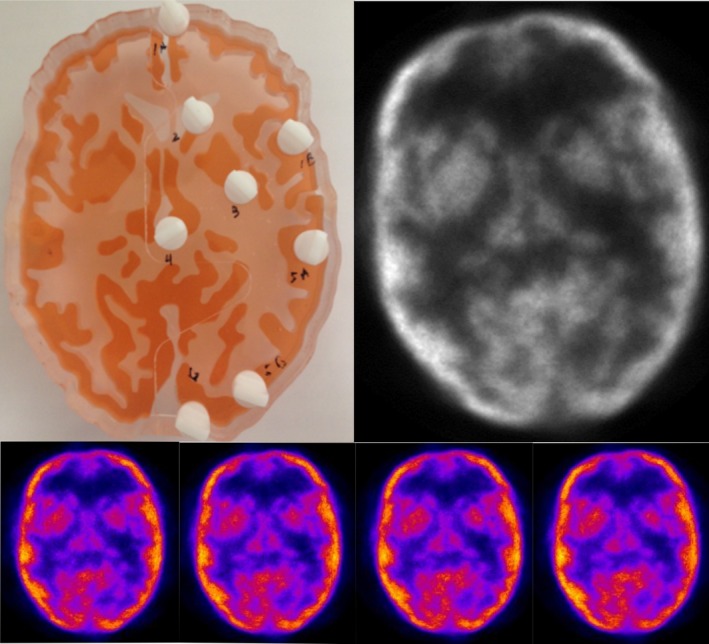
Top Left: The actual multicompartmental phantom used for imaging. Top Right: A 12‐mm reconstructed slice of the phantom. The phantom was reconstructed in 1‐mm slices, and then grouped together to form a 12‐mm slice. Bottom: Four consecutive central 1‐mm phantom slices

In addition, the grey matter compartment of the Hoffmann phantom was equally filled with a low level of F^18^‐FDG tracer (less than 100 μCuries) and imaged for 4 hr. Reconstructed images were iterated 10 times and binned into 1‐mm slices. The images were displayed in ImageJ (Fig. 3).

### 2.6 Human imaging

We imaged four consenting patients under approved IRB (institutional review board) protocol, which followed patient imaging with whole‐body mCT PET/CT from Siemens (Jakoby et al., 2011). The patients (all male) were cancer patients requiring clinical whole‐body PET/CT. In our medical center an additional brain scan is a part of the routine diagnostic PET/CT protocol. Imaging with Helmet_PET was performed after PET/CT scans and was divided into four components: (1) a fast 30 s scan, (2) a 3 min scan, (3) a 10 min scan, and (4) a 1 min scan with the patient intentionally turning his head left and right by an angle of about 45 degrees at a slow pace according to their own volition. The last 1 min scan was intended to demonstrate that imaging can be performed while the patient's head is moving in a limited but unrestricted way.

Two of the four patients were imaged immediately following the PET/CT scan, and two after over 4 hr of wait time (with lowered activity, primarily due to radioactive decay of F^18‐^FDG) following the whole‐body PET/CT scan.

Even though we did not predict equivalency due to the prototype nature of the device and the timing and dose differences between the scans, we performed quantitative ROI measurements of activity in multiple brain regions to test whether the brain regions showed a similar pattern of increased versus decreased activity as with those obtained via the standard PET images of the same participants. Neuroanatomy‐based ROIs were guided and confirmed by a practicing radiologist. ROIs were coregistered by the program and compared between Helmet_PET and PET/CT. The values reported in our results are mean voxel value of the ROIs divided by the mean voxel value of the entire slice (whole brain – 2D), resulting in the percentage of mean ROI value compared to mean whole slice value.

### 2.7 Ethical statement

This study was approved by the local institutional review board (IRB) at WVU. Prior to participation, all patients were informed about the nature of the study, consented to participation in the study, and signed an informed consent form. Patients were compensated with 50 dollars for their cooperation, inconvenience, and extra costs incurred.

## 3 Results

### 3.1 Phantom images

The phantom showed a uniform level of activity across all areas, which is consistent with how the phantom was filled. In addition, both the 15‐mm and 1‐mm slices showed neither artifacts nor abnormalities, and the reconstructed image pattern was very similar to the pattern dictated by the physical phantom (Fig. 3).

### 3.2 Patient images

As the first two patients were scanned closer in time to the initial injection, this activity turned out to be too high for our imager (saturating its performance). The patient data from the two patients waiting 4 hr for reduced F^18‐^FDG activity produced usable results that we present here. We thus learned that low dose is not only possible but necessary with the current prototype, although changes in how the applied readout electronics may be able to accommodate the pulse pileups from high doses needs to be addressed in future models.

**Figure 4:**
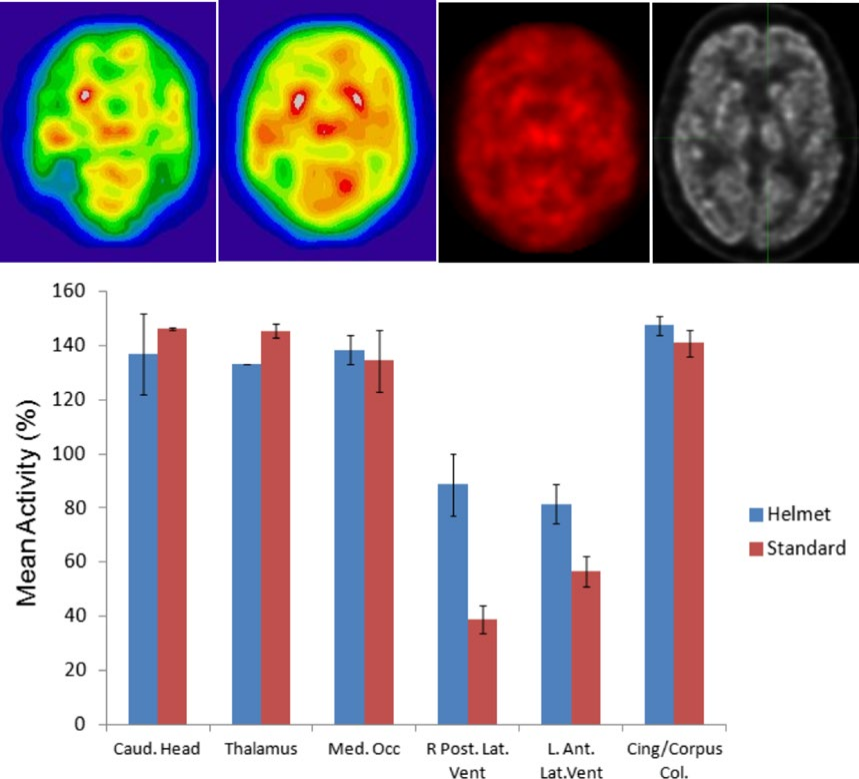
Top Left: One min PET scan with the head turning back and forth about 45 degrees (Patient 1). Image resolution after software filtering is about 1 cm in this case, and slice thickness is 4 mm. Top Left Center: 10 min PET scan while the patient remains still (Patient 1). Spatial resolution and slice thickness are the same as in the top left panel. Top Right Center: Helmet_PET image of the brain once imported into the MIM software (Patient 1). For comparison purposes, this image is 2‐mm thick with greater than 4‐mm spatial resolution. Top Right: The same slice of the participant as in the top right center panel, but using the clinical Siemens mCT PET/CT system. Bottom: Six different ROIs which were compared between the Helmet_PET images and the clinical PET/CT images for two patients. The comparison is expressed as the mean voxel value of the specified ROI divided by the mean voxel value of the whole slice, resulting in the reported percentages. The first five ROIs were two dimensional from 2‐mm brain slices. The final ROI was an experimental test of a three‐dimensional ROI encompassing a portion of the medial cingulate cortex. It is worth noting that the three‐dimensional ROI was still normalized to a 2D longitudinal slice, just as in the two‐dimensional ROIs

Two patients included in the analysis were imaged with Helmet_PET more than 4 hr after their clinical PET/CT scan. The obtained images were then compared with the PET/CT images of the patient's head. We estimate, based on the half‐life of F^18‐^FDG (~110 min) that the activity level after the 4 hr wait period in the second two patients was 25% the amount during their clinical scan. The selected results of this comparison are presented in Figure 4.

### 3.3 ROI Comparison

Several ROIs were drawn for this experiment to perform a quantitative analysis. Overall, as in typical resting‐state F^18‐^FDG ‐PET images, we found that the highest metabolism in both Helmet_PET and standard PET images was the basal ganglia, thalamus, and occipital lobe. Helmet_PET also showed a similar metabolic signal compared to standard PET at a corpus callosum/cingulate gyrus ROI, although our prototype imager overestimated the activity of the ventricles (Fig. 4). However, the measurements for the two scans were performed at times shifted by 4 hr, so signal differences may be the reflection of an actual change.

## 4 Discussion

We propose here that light‐weight freely wearable low‐dose brain PET imagers could be very useful in research and clinical applications, and demonstrate this concept with a rudimentary imager. Our custom‐built imager moved with the head, and was able to collect low‐dose images of upright participants with active head motion that showed the typical resting pattern of F^18‐^FDG activity. Although lower in resolution due to the early prototype design, the classic resting‐state brain signature was observed, including elevated activity in midline structures, basal ganglia, and the thalamus, even during large deliberate head movements while wearing the Helmet_PET. To our knowledge, these results are the first to demonstrate the feasibility of a wearable PET imager using human subjects.

### 4.1 Considerations related to motion tolerance

The unique advantage of a wearable PET imager is the ability to acquire images during motion that does not significantly disrupt the imaging. In our proof‐of‐concept study, during a 1‐min active image acquisition, the Helmet_PET collected brain images showing the typical resting‐state pattern comparable to a 10 min stationary scan. Image acquisition during head turning, still far removed from strenuous physical activity, is a significant advancement in human mobile molecular imaging. The mechanical support was rudimentary, and a full‐sized wearable PET imager would require a more sophisticated and robust support to enable a heavier device with increased freedom of movement. As the current prototype was approximately 6 lbs, we predict that an imager with full brain coverage would be about 20 lbs using conventional geometries. Accompanying safety mechanics will need to be designed, with weight‐compensation included. Providing the appropriate support mechanics to both lift this weight from the participant while at the same time encouraging natural movement will remain a challenge in future development.

Trade‐offs between weight, mobility, full brain coverage, sensitivity, and resolution will have to be considered for future wearable designs. Other methods for addressing the weight associated with full brain coverage may benefit from implementing advanced geometries such as detectors placed under the chin to increase capture of annihilation photons emitted from the bottom of the brain (Tashima, Ito, & Yamaya, 2013; Yamaya et al., 2015). Novel geometries would provide higher quality imaging and also could reduce the total number of detector modules required.

### 4.2 Considerations for radioligand imaging

An exciting feature of a wearable PET and other head‐only PET devices is the acquisition of brain images with a lower level of radioactivity. In the experiments with the Helmet_PET prototype, the lower dose requirement was demonstrated by image acquisition 4 hr after the initial injection, giving us the estimate that at least 25% of the standard dose for the human participants would be sufficient. The phantom‐based studies also supported the ability to image with lower doses (imaged at less than 100 μCuries in the field of view). The method of waiting for the clinical dose activity to subside may have led to shifts in the F^18‐^FDG pattern, and although not ideal, had the advantage of being completely noninvasive for this proof‐of concept study justifying future human studies that start with a 25% or even 10% dose injection.

The low‐dose feature has several major advantages, making wearable PET devices a more palatable tool for both patient and nonpatient research studies, and easier to justify the cost/benefit ratio with a much lower risk to participants and staff. Longitudinal studies would be enabled; allowing one to monitor brain activity changes over time, such as with behavioral medicine treatments, disease progression, or other therapeutic techniques. Clinically, the low dose would be especially critical for children requiring head‐only PET (such as epilepsy patients) and other neurological and psychological disorders common in children, such as autistic spectrum disorders.

Functional imaging during behavioral and movement tasks may benefit from increased temporal resolution of repeated O^15^‐H_2_O injections. Also promising is dynamic imaging with ligands with longer half‐lives, such as C^11^ and F^18^ compounds. For example, in a study using RatCap, researchers were able to correlate the rat level of behavioral activity with the difference in free versus bound C^11^‐Raclopride in the striatum (Schulz et al., 2011), and in a standard PET study in humans, 5 min temporal resolution with F^18^‐FDG was obtained during a visual stimulus task (Villien et al., 2014). Real‐time task‐related imaging enabled by a wearable PET and higher temporal resolution techniques may constitute an improved functional PET (fPET) able to image the types of activities off‐limits to fMRI.

### 4.3 Considerations in imager technology

The early prototype Helmet_PET was only designed to demonstrate and test feasibility of the wearable PET concept, and was thus missing some of the elements a research or clinically relevant device would require. For example, as we lacked sophisticated detector response corrections and therefore made use of an effective, but not ideal “flood correction” for image normalization (see Methods). System modeling would be recommended for normalization in a next generation device. In addition, the length of the signal cable used was longer than required for a stand‐alone device as it was also designed to test MRI compatibility of the device, the promising results of which will be reported separately (Bauer et al., 2013). The fact that this device was also designed for MRI compatibility meant that the amplifiers were also placed at a distance from the SiPMs (to reduce interference in the magnet), thus further introducing noise into the system in the upright application. Furthermore, major design elements including now standard corrections such as protection against scattered radiation, parallax distortion, and correction for random coincidences were omitted in this pilot design, and also contributed to noisy images that required blurring in the image processing software. Unlike the phantom studies in which up to 2‐mm resolution could be observed, other factors such as the radiation outside the field of view from the body of the participants as expected resulted in elevated noise in the images. We will analyze ways of adding shielding with minimal contribution to device weight.

Based on the images produced by Helmet_PET, we would recommend that the diameter of a future wearable imager be increased from 21 cm to ~25 cm. In the current prototype, the spatial resolution decreased substantially at the periphery versus the center region, and portions of the cortex may have been outside the field of view of the imager, both effects due to very tight geometry. Loss of spatial resolution toward the edge of the imager could be partially mitigated with the introduction of depth of interaction (DOI) correction (Gong et al., 2016).

Finally, there are modeling and software optimizations that would benefit a next‐generation system. One limitation of the current prototype is rate capability performance. This particular issue, along with faster acquisition software, should be considered in future implementations. Also, improved software corrections such as improved brain registration with clinical software, faster reconstruction algorithms, and system modeling similar to that developed by Yamaya et al. (2008) would constitute a significant advancement.

### 4.4 General limitations of a wearable PET concept

Certain limitations of a wearable PET imager will persist, even with the most sophisticated device. Even though wearable PET devices would allow for head and body motion, there would still be some movement limitations. In cases where the imager is large and bulky, even with support mechanics, very rapid head movements would be limited due to the inertia of heavy equipment attached to the head. The issue of the weight of the imager and the support mechanics are further complicated when considering shielding to prevent interference from radiation emitted from the body. As such, these trade‐offs would need to be considered based on research and clinical need and whether the main priority is image quality or participant mobility. Most recently implementation of Time of Flight (TOF) capability in brain imaging is discussed and analyzed in simulations as a method to improve S/N ratio, reduce the background effects, including scatter of emissions from the body without introducing shields, and effectively to increase sensitivity even with less scintillator material and lower total weight of the imager (Gong et al., 2016).

## 5 Summary and future directions

We have introduced, presented, and discussed the concept of a wearable PET imager using a prototype light‐weight brain‐dedicated upright device. We demonstrated that imaging with even a very simple ring of compact detectors could produce a similar pattern to the standard clinical PET/CT, but unlike images obtained using standard methodology, the patients could move their head with a Helmet_PET type imager. We expect that the next‐generation device will yield a significant improvement in image quality as well as more functional and versatile mechanical support, eventually including the ability of the participants to stand and walk with the imager attached to the head. We have plans for several iterations of next‐generation devices (Bauer, Brefczynski‐Lewis, Lewis, Mandich, & Majewski, 2014; Brefczynski‐Lewis, Bauer, Lewis, Mandich, & Majewski, 2014; Gong et al., 2016; Harrison et al., 2015; Kinahan et al., 2015) to further explore our theoretical concept. Ultimately, we envision multiple designs that could be focused either on full brain coverage and high efficiency with the drawback of less mobility, or more on enabling free movement at the expense of full coverage or maximum sensitivity.

We plan to develop a brain PET system which not only has high performance but is also safe for the patient, comfortable, and flexible, as well as robust and economical in order to promote its widespread use. Ideally, this will allow subjects and patients to receive low‐dose imaging brain scans in more accessible locations such as in psychiatric and stroke clinics, as well as imaging patients who have difficulty remaining still. A wearable PET imager that allows seated motion of the head and body would be a remarkable advance in our ability to study natural behaviors such as upright reaching and postural control, social interactions, and imaging of patient populations who are unable to remain still without sedation such as in Parkinson's, Alzheimer's Disease, as well as individuals with ASD or ADHD. Ultimately we envision potential applications in the ambulatory environment in which insight into the functioning of the healthy and diseased human brain during active tasks such as physical therapy, tasks in more controlled virtual reality environments, or even more natural environments outside the laboratory can be ascertained.

## Funding Information

WVU_CTSI (Grant/Award Number: ‘U546M104942'), NIH Cobre Sub Award (Grant/Award Number: ‘NIH P30 GM103503') NIMH R24MH106057.

## Conflict of Interest

U.S. patent held by Dr. Stan Majewski (Majewski & Proffitt, 2011).
